# Feline Neonatal Isoerythrolysis and the Importance of Feline Blood Types

**DOI:** 10.4061/2010/753726

**Published:** 2010-06-02

**Authors:** Ana C. Silvestre-Ferreira, Josep Pastor

**Affiliations:** ^1^Department of Veterinary Sciences, Veterinary Hospital, University of Trás-os-Montes e Alto Douro, 5001-801 Vila Real, Portugal; ^2^Department of Animal Medicine and Surgery, Veterinary Faculty, Autonomous University of Barcelona, 08193 Bellaterra, Spain

## Abstract

Although feline neonatal isoerythrolysis is rare, associated mortality rate is high. It results from mating of type B blood queens with type A or AB blood toms. A comprehensive review on feline blood types and feline neonatal isoerythrolysis physiopathology, clinical features, diagnosis, treatment, and prevention is covered.

## 1. Introduction

Kitten death in the neonatal period is frequent in cat breeders [1]. Perinatal death in the first two weeks is generally associated with neonatal isoerythrolysis (NI) emaciation, congenital abnormalities, hypoglycemia, hypothermia, low weight at birth, problems during labor, ambient factors, factors related to the mother, and neonatal infections [1, 2]. In the UK, a survey on kitten mortality (from birth to 16 weeks of age), revealed that the majority of deaths in the perinatal period (< one day) was due to NI [3]. Neonatal isoerythrolysis is believed to be a major cause of fading kitten syndrome [4]. The fading kitten syndrome is a common cause of death in the first weeks of life. It is a poorly defined syndrome, characterized by anorexia, lethargy and emaciation [1, 2].

Neonatal isoerythrolysis, or neonate hemolytic disease is a disease of humans and domestic animals and has been observed in cats, horses, pigs, dogs and cows. It is characterized by immune destruction of red blood cells [5]. Neonatal isoerythrolysis differs in domestic animals and humans in the fact that the syndrome is revealed at the postpartum stage in animals, and during embryogenesis in humans. Natural occurrence of NI is recognized in horses [5], cats [5–8], as well as in humans, but has been rarely identified in other species where it takes place after blood transfusions, vaccination, or previous pregnancy [5].

## 2. Feline Blood Groups

Cats have one blood group, the feline AB blood group system that is characterized predominantly by two blood types: type A, the most common, and type B. A third blood type is also known the rare AB [9]. Blood types are inherited as a simple autosomal Mendelian trait, with A being dominant over B. Type A blood cats may have *AA*, or *Ab* genotype. Type B cats are always homozygote. Little is known about the type AB inheritance mode which seems to be a third allele, or a case of codominance [4, 10, 11]. Although previous studies have not been definitive about the inheritance of type AB, a new study indicates that AB is allelic to A and B in cats represented as *A* > *a* ^*ab*^ > *b*. Possible genotypes/phenotypes would be *AA *(Type A); *A* *a* ^*ab*^ (Type A); *Ab* (Type A); *a* ^*ab*^ *b* (Type AB), and *a* ^*ab*^ *a* ^*ab*^ (Type AB), and *bb* (Type B) [12]. Feline neonatal isoerythrolysis (FNI) appears when type B mothers mate with type A tomcats [1, 5, 6]. Crosses between type B cats only produce type B kittens [4]. An important characteristic of the feline AB blood group system is the presence of naturally occurring alloantibodies against the blood type they lack. Natural means that there is no need for previous exposition to blood or blood products. All type B cats aged more than three months possess high-titer naturally occurring anti-A alloantibodies with haemolysing and haemagglutinating activity, but not all of type A cats present measurable titers of naturally occurring anti-B alloantibodies (Table 1). In type A cats, naturally occurring anti-B alloantibodies have a feeble haemolysing and haemagglutinating activity. Type AB cats do not possess any kind of anti-AB alloantibodies [4, 9, 13]. Recently, the presence of a new alloantibody produced against a common red cell antigen was described and termed as Mik. The clinical relevance of anti-Mik alloantibodies was described as an acute hemolytic transfusion reaction after inadvertent transfusion of Mik-positive blood to the Mik-negative renal transplant recipient [14].

Alloantibodies titers may suffer geographical variation (Table 1); this hypothesis is supported by data from the UK, Portugal, Spain, and Turkey where lower anti-A antibodies titers than those previously reported were found in type B cats [15–19].

The naturally occurring anti-A alloantibodies present in type B cats are responsible for FNI as well as severe red cell destruction in mismatched blood transfusions in a manner that inclusively primiparous queens may present FNI litters [1, 6, 7].

Prevalence of FNI is unknown but it varies according to the number of type B cats in a given population. Feline blood types in nonpedigree cats vary geographically (Table 2). Frequency of blood types also varies among breeds (Table 3), but breed variation is not affected geographically [20–22]. 

Breeds like Siamese, or those genetically related, only present type A cats so the risk of FNI occurrence is null. Others, like British Shorthair, Devon Rex, Persian, Abyssinian, Turkish Angora, and Turkish Van, present type B cat frequencies that vary between 10% and 60% [21, 23]. Random mates are at a great risk of FNI in these breeds. 

The gene frequencies for A and B alleles can be estimated in certain breeds by using the Hardy-Weinberg equilibrium. Assuming random mating, *q* ^2^ is the proportion of type B cats; *q* = frequency of B allele (recessive). As for dominant allele A it corresponds to *p* = 1 − *q*; FNI = (*p* ^2^)(*q* ^2^) + 2*p* *q*(*q* ^2^). Recently, Malik and coworkers [20] in Sidney, Australia and Arikan and coworkers [24] in Turkey, estimated, for the nonpedigree cat population, the proportion of random mating at risk of developing neonatal isoerythrolysis to be 23% and 18.6% respectively. Previously, Bücheler [25] described the risks of incompatible mating as between 14% and 25% for Persian and Abyssinian populations.

## 3. Feline Neonatal Isoerythrolysis Physiopathology

Feline placenta is of endotheliochorial type [6, 38]. Chorionic endothelium is closely linked to maternal capillary endothelium [38]. It only allows a small and insignificant passage of maternal antibodies, 5 to 10% [6, 38]. Kittens get maternal antibodies, IgG in most cases, by suckling colostrum during the first days of life [6, 7]. The development of the immune system is a critical period for the kitten. In this period, maternal immunity is an important factor, but in some cases it can also cause disease. Kittens start to produce their own alloantibodies soon after birth, reaching their maximum level in the first months of life while the level of maternal antibodies is low at 6 to 8 weeks [25, 39]. FNI affects the A, or AB blood type kitten, born from a B blood type mother by getting anti-A antibodies when it starts suckling [1, 6, 7].

## 4. Clinical Features

FNI clinical features depend on haemolysis grade and severity. Suckling colostrum allows the passage of naturally occurring alloantibodies from mother to the neonate. Antibodies recognize the antigenic determinants in the kitten red cell surface, causing intra or extravascular haemolysis. Extravascular haemolysis can occur in the spleen or liver. Haemolysis leads to anemia, nephropathy, or disseminated intravascular coagulation [1, 7]. Determinants to the degree of haemolysis or severity are still unknown, but the large variation in clinical signs within a litter suggests differences in colostral antibody uptake as a determinant factor [1, 40]. Generally, kittens are born healthy and nurse energetically, but after colostrum ingestion, clinical signs appear in a few hours or days. Some may die in a few hours without presenting any kind of clinical signs. Others stop suckling in the first days of life and fade. The key signs to diagnose FNI are dark red-brown urine, indicating severe intravascular haemolysis and haemoglobinuria, but they may also present jaundice, anemia, and weakness with death occurring in the first week of life. Secondary clinical signs are pale mucous membranes and those related to decreased oxygenation: lethargy, tachycardia, tachypnea, collapse, and death. Hypoglycemia and metabolic acidosis may be present associated to stopped or decreased suckling [1]. Those who survive may develop tail tip necrosis (Table 4) [1, 7, 8].

Tail tip necrosis is associated with cold IgM action, with haemagglutination, clot formation, and ischemic necrosis. In adults, due to other etiologies, ears, paws, nose, scrotum, and tail tip are usual sites of action for cold agglutinins, but in kittens, protected by the queen's body heat and because ears are folded against the head, these sites are protected; consequently tail tip in neonatal kitten is the most vulnerable site for IgM action [8].

## 5. Diagnosis

**Figure 1:**
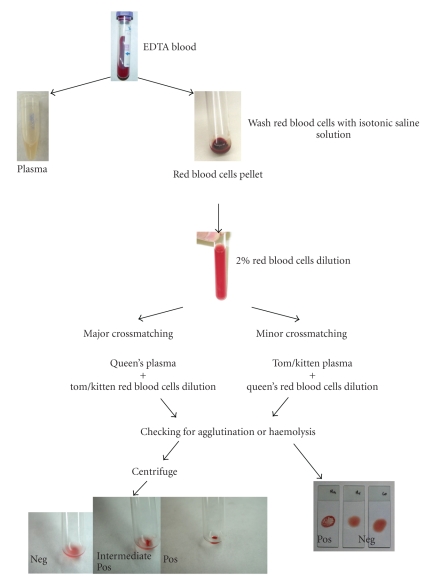
Crossmatching schematic representation. See Table 5 for procedure explanations. Crossmatching can also be performed as a slide test by using the same protocol. Haemolysis can better be recognized in the tube test when compared to slide method.

Diagnosis is performed on the basis of clinical signs and confirmed by blood typing the queen and the kitten. If blood typing is not possible, a blood crossmatching can be performed. (Table 5; Figure 1). Kittens with FNI present a positive Coomb's test [1] which confirms the immune-mediated nature of this process. 

If FNI is suspected all kittens should be blood typed. At birth, cord blood from the placenta may be used to type kittens. 

The crossmatching checks for serologic compatibility or incompatibility and it may be possible to detect any incompatibility, even outside the AB system. This is an important advantage compared to blood typing that only recognizes blood type antigens [21]. When an incompatible major crossmatching between the queen and the kitten is found, FNI may be suspected. 

Nowadays for blood typing purpose, besides the traditional method, there are different commercially available methods: the card test (DMS Laboratories Inc, Flexmington, New Jersey), and the gel column technique (DiaMed AG, Cressier Sur Morat, Switzerland) [42]. Recently, two companies, in Japan and France, have introduced two novel techniques: a tube test (Shigeta Animal Pharmaceuticals Inc, Oyabe City, Japan), and a new immunochromatographic cartridge (Alvedia, Lyon, France) [42, 43].

Most recently researchers from UC Davis have found the gene associated with the B blood group and its mutation. They developed a diagnostic DNA test so that animals can be tested at an early age from a buccal swab. The genetic test for the cat blood group identifies the recessive *b* allele which is associated with the B serotype. This test has not been fully validated in the Ragdoll and Turkish Angora breeds, because, in some animals, results from DNA and serological tests are not concordant, but cat breeders can greatly benefit from this test for selection of mating pairs. 

Necropsy is an important step in FNI and perinatal death diagnosis. As death may occur in different stages of the disease, pathological findings depend on the moment of death. The bladder may be filled with dark red-brown urine and precipitated haemoglobin. The body may appear icteric and the spleen enlarged. The spleen and liver may present marked erythrophagocytosis and extramedullar haematopoiesis. In the kidneys large red-orange tubular casts compatible with haemoglobin or acute tubular necrosis may be seen. Systemic effects of immune-mediated haemolysis, disseminated intravascular coagulation, anemia, and acute renal failure [1, 5], are the apparent cause of death in kittens suffering from FNI.

## 6. Treatment

FNI treatment should be aggressive and immediate. FNI treatment steps comprise replacement of passive immune protection, a blood transfusion if clinical conditions deteriorate, and life support treatment. When the first clinical signs appear, type A or AB kittens should be immediately removed from their mothers, as long as they continue to suckle more anti-A immunoglobulins they receive [1, 7]. Type B kitten can continue to suckle. Kittens should receive immunoglobulin-rich colostrum during the first 12 hours of life to obtain optimal serum antibody titers and acquire adequate passive immune protection throughout the neonate period [7]. The interval of transfer of maternal antibodies seems to be of only 16 hours. The IgG are absorbed by the neonate during this period [1, 7]. After this, intestinal mucosa loses its permeability, and even when administered, immunoglobulins are not absorbed [7]. Therefore, kittens only need to be removed from their mothers for the first 16 to 24 hours of life [1, 7]. Kittens may be fed with a commercial milk replacer, previously frozen milk from a type A blood mother, or be placed with a foster type A blood queen [1, 7]. Prevention of passive immunity failure will be addressed later on.

**Figure 2:**
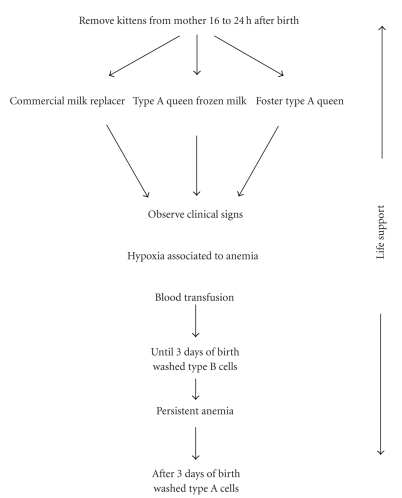
Schematic representation of feline neonatal isoerythrolysis treatment. To wash red blood cells, 2 to 3 mL donor blood should be collected into EDTA and centrifuged; the supernatant should then be discarded. The blood is restored with isotonic saline solution into twice its volume and again centrifuged. After discarding supernatant, repeat this action and dilute cells to transfusion with an equal saline volume.

If anemia is severe and becomes worse, a blood transfusion should be considered. Kittens severely anemic with hypoxia signs should receive 2 to 3 mL of previously washed blood cells during the first 3 days of life (Figure 2). Blood donor selection is the key to a successful transfusion. 

In an A or AB blood kitten with FNI, the queen's circulating colostral antibodies are anti-A. Transfusion of type A cells simply adds more vulnerable cells to the kitten's circulation. The best blood donor would be the queen as she cannot obviously react to her own antibodies [1, 44]. 

Blood can be transfused via a spinal needle into the trochanteric fossa.^1^ In this way, about 90% of red blood cells are in the blood stream in 10 minutes [1, 44–46]. Due to the shortened life span of transfused red cells and a continued destruction of the kitten's own cells, anemia may worsen and a new blood transfusion may be essential. The kitten starts form its own anti-B alloantibodies soon after birth, and maternal antibodies from colostrum start to decline. Therefore, if another blood transfusion is required after 3 days postpartum, a washed type A blood administration should be considered [1]. After anemia is corrected, the kitten should receive life support treatment associated with electrolytic changes and tissular hypoxia. Even when kittens are removed from their mothers, as soon as the first clinical signs become present, the mortality rate associated with FNI is high, making prevention the most important step [1, 7].

## 7. Prevention

The best method to prevent FNI is to avoid incompatible mating between type B blood queens and type A blood toms. Knowledge of the parent's blood type is essential for FNI prevention. To assure blood compatibility, blood typing might be done with an in-house blood typing card, gel, or tube test, that appear to be reliable clinical laboratory methods for feline blood typing [15, 42], or with crossmatching [1, 7, 8] (Table 5; Figure 1). If there is a need to mate a type B queen with a type A tom, the best way to prevent FNI is to remove the kitten from the mother for 24 hours preventing them from nursing colostrum [7, 8] (Table 6). Failure in passive immunity might be solved by using previously frozen colostrum, from another queen's milk [6, 7]. 

In most mammalian species, the immunoglobulin concentration in colostrum is generally much higher than that in milk [47]. Although previous studies show that milk immunoglobulin concentration in queens is similar to that present in colostrum [6, 7], nowadays we know that cats have both colostral and milk phases of lactation distinguished by the concentration of IgG and IgA [48]. In cats, IgG and IgA colostrum concentration is greater than that in serum, but reduced concentrations of IgG and IgA have been demonstrated in milk relative to colostrum [48]. IgM concentrations are lower in colostrum and milk than in the queens' serum [6, 7]. 

Although transplacentary immunoglobulin transfer is more efficient for IgG [40], fostering on queens in mid-lactation does not provide protective concentrations of immunoglobulins in colostrum deprived kittens [48]. A previous study also shows that parenteral administration of 150 mL/Kg of adult feline serum results in a normal concentration of IgG in colostrum deprived kittens. Serum donors must be blood typed [49].

Another study revealed that in vitro neutrophilic and plasmatic activity hosts a defense against bacterial and other microorganisms, which is similar in kittens suckling colostrum and in those that do not suckle [50].

Because maternal IgG levels have a short life span—approximately 4.4 days (IgG life span is shorter in kittens than in puppies) [6] and the onset of IgG and IgA production is late (IgG production starts by the 5th to 6th week of age and IgA shortly after, while, in contrast, IgM steadily increases to a plateau on the 60th day of life) [6, 51], kittens are vulnerable between the third and fourth week of life. For kittens that have been deprived of colostrum, early vaccination is recommended whenever there is a risk of viral infection [7].

In conclusion, FNI is rare but the mortality rate is high. It results from random mating between type B blood queens and type A or AB blood toms. The best way to prevent FNI is to blood type progenitors, mostly those belonging to breeds with high incidence of animals with type B blood. 

## Figures and Tables

**Table 1:** Minimum anti-A antibodies titers presented by type B cats and percentage of type A cats presenting anti-B antibodies. In all studies, all type B cats presented anti-A antibodies titers. Anti-B antibodies in type A animals ranged from 1 : 2 to 1 : 16 in most cases.

COUNTRY	Minimum titers of anti-A antibodies presented by type B cats	% of type A cats presenting anti-B antibodies
USA [25]	1 : 64	36
Australia [13]	1 : 8	35
Turkey		
Pedigree [17]	<1 : 4	60.6
Nonpedigree [19]	<1 : 4	70
Portugal [18]	1 : 16	12.5
Spain (Gran Canary) [16]	1 : 16	24.4
United Kingdom [15]	1 : 4	44.3

**Table 2:** Feline blood types geographical distribution.

Country	Type A (%)	Type B (%)	Type AB (%)
Austria [26]	88	12	—
Finland [26]	100	—	—
Holland [26]	95.8	4.2	—
Scotland [26]	97.1	2.9	—
Denmark [27]			
*Nonpedigree*	98.1	1.9	—
*Pedigree*	89.2	10.8	—
Spain			
Barcelona [28]	94	4	2
Gran Canária [16]	85.9	9.4	4.7
France [29]	85	15	—
Greece [30]	78.3	20.3	1.4
Germany [31]	93.9	5.4	0.7
*Pedigree*	83.3	14.9	1.8
Hungary [32]			
*Nonpedigree*	100	—	—
*Pedigree*	84.2	15.8	—
Italy [33]	87.1	12.9	—
Portugal [18]	90.3	3.8	5.9
Switzerland [34]	99.6	0.4	—
United Kingdom [15]			
*Nonpedigree*	54.6	40.1	5.3
*Pedigree*	87.1	7.9	5.0
Turkey [24]			
*Nonpedigree*	73.1	24.6	2.3
Australia			
*Nonpedigree*			
(Sidney [20])	62	36	1.6
(Brisbane [9])	73.3	26.3	0.4
Japan [35]	90.3	9.7	—
USA [36]	98.1	1.7	0.1
*Nonpedigree* [37]	99.6	0.4	—

**Table 3:** Breed distribution of feline blood types; *breeds with reported type AB cats (Adapted from Giger [36] and Arikan and coworkers [38]).

Breed	Type A %	Type B %
Abyssinian	84	16
American shorthair	100	0
Birman*	82	18
British shorthair*	64	36
Burmese	100	0
Cornish Rex	67	33
Devon Rex	59	41
Exotic shorthair	73	27
Himalayan	94	6
Japanese Bobtail	84	16
Maine Coon	97	3
Norwegian Forest	93	7
Oriental shorthair	100	0
Persian	86	14
Scottish fold*	81	19
Siamese	100	0
Somali*	82	18
Sphinx*	83	17
Tonkinese	100	0
Turkish Angora	54	46
Turkish Van	40	60

**Table 4:** Key signs to FNI diagnosis.

Reaction	Signs
Unspecific	Stop suckling → fade
	Sudden death
Severe	Haemoglobinuria
	Jaundice
	Anemia
	Weakness → death
	Decreased oxygenation
	Lethargy
	Tachycardia
	Tachypnea
	Collapse → death
	Altered nutrition
	Hypoglycemia
	Metabolic acidosis

Survivors	Tail tip necrosis

**Table 5:** Crossmatching protocol. Major crossmatching should be compatible at 37° and 24°C (cold agglutinins) and minor at 37°C (Adapted from Fox [41]).

1. Collect 2 mL of blood into EDTA from tom/kitten and queen.

2. Centrifuge 3400 × g 1 minute, separate plasma from red blood cells. Keep plasma.

3. Wash red blood cells two times, into at least twice its volume, with isotonic saline solution.
Discard supernatant and keep red blood cells.

4. Dilute red blood cells at 2% : 10 *μ*L washed red blood cells plus 490 *μ*L isotonic saline solution.

5. Major crossmatching:
2 drops of (50 *μ*L) tom/kitten's red blood cell dilution
2 drops of (50 *μ*L) queen's plasma

6. Minor crossmatching:
2 drops of (50 *μ*L) queen's red blood cell dilution
2 drops of (50 *μ*L) tom/kitten's plasma

7. Negative control:
2 drops of (50 *μ*L) tom/kitten's red blood cell dilution
2 drops of (50 *μ*L) tom/kitten's plasma

8. Incubate 30 minutes at 25°C and also at 37° and 24°C.

9. Centrifuge 3400 × g 1 minute.

10. Examine the supernatant for any haemolysis. Any haemolysis indicates also incompatibility.

11. Rotate tubes between the fingers to mix and examine for agglutination. The presence of agglutination indicates a positive test and
tom or kitten/queen incompatibility.

**Table 6:** Fundamental steps for feline neonatal isoerythrolysis prevention.

To know progenitors blood types by the use of blood typing and/or crossmatching.
To avoid mates between type B queens and type A toms, or not to mate type B queens.
Kittens born from mates between type B queens and type A toms should be removed from their mother on the first 24 hours of life.

## Endnotes

1. Cannula placement in the trochanteric fossa of the femur. Spinal needles according to animal size may be used. Neonates have soft spongy bones that can be penetrated with 18 to 25 gauge hypodermic needles. Aseptic technique must be used in preparing the access site. Make a small incision in the skin over the insertion site. For placement in the trochanteric fossa of the femur, the needle should be maneuvered off the medial aspect of the great trochanter into the trochanteric fossa. To avoid damaging the sciatic nerve, the coxofemural joint should remain in a neutral to slightly extended position with the femur externally rotated, during the placement of the cannula. Following the insertion of the cannula through the skin, pressure is applied to the needle along with firm rotation movements [45]. The stylet inside the cannula should not be removed until the blood administration starts. The stylet prevents a core of bone from obstructing the lumen during insertion [46].
